# Enhancing UWB Indoor Positioning Accuracy through Improved Snake Search Algorithm for NLOS/LOS Signal Classification

**DOI:** 10.3390/s24154917

**Published:** 2024-07-29

**Authors:** Fang Wang, Lingqiao Shui, Hai Tang, Zhe Wei

**Affiliations:** 1 School of Science, Civil Aviation Flight University of China, Guanghan 618307, China; cafuc_wf@foxmail.com; 2 School of Computer Science, Civil Aviation Flight University of China, Guanghan 618307, China; shuilingqiao23@cafuc.edu.cn (L.S.); findz@cafuc.edu.cn (Z.W.)

**Keywords:** UWB, NLOS error, NLOS identification, chaotic mapping, BP neural network

## Abstract

Non-line-of-sight (NLOS) errors significantly impact the accuracy of ultra-wideband (UWB) indoor positioning, posing a major barrier to its advancement. This study addresses the challenge of effectively distinguishing line-of-sight (LOS) from NLOS signals to enhance UWB positioning accuracy. Unlike existing research that focuses on optimizing deep learning network structures, our approach emphasizes the optimization of model parameters. We introduce a chaotic map for the initialization of the population and integrate a subtraction-average-based optimizer with a dynamic exploration probability to enhance the Snake Search Algorithm (SSA). This improved SSA optimizes the initial weights and thresholds of backpropagation (BP) neural networks for signal classification. Comparative evaluations with BP, Particle Swarm Optimizer–BP (PSO-BP), and Snake Optimizer–PB (SO-BP) models—performed using three performance metrics—demonstrate that our LTSSO-BP model achieves superior stability and accuracy, with classification accuracy, recall, and F1 score values of 90%, 91.41%, and 90.25%, respectively.

## 1. Introduction

With the proliferation of the Internet of Things (IoT) and smart devices, the demand for indoor location services across various industries is increasing. Consequently, indoor positioning technology has become crucial. This technology not only provides precise spatial data for intelligent scenarios but also offers real-time dynamic tracking capabilities, significantly enhancing user experiences and operational efficiency. Among the many indoor positioning technologies, ultra-wideband (UWB) applications have garnered widespread attention due to their high accuracy, low power consumption, and reliable signal transmission capabilities [1]. However, in practical applications, non-line-of-sight (NLOS) propagation has a significant negative impact on the accuracy of UWB indoor positioning [2]. Under NLOS conditions, signal transmission is obstructed and reflected by walls, furniture, or other obstacles, leading to extended and distorted signal paths. This results in increased signal arrival times, causing a positive bias in distance measurement and significantly reducing positioning accuracy. Therefore, to improve the accuracy of UWB indoor positioning, it is essential to identify NLOS signals before positioning [3].

Regarding the phenomenon of non-line-of-sight (NLOS) propagation, current methods for identifying NLOS and line-of-sight (LOS) signals can be mainly categorized into three types. The first type is based on distance estimation. This method distinguishes LOS and NLOS signals by analyzing the probability density function of measurement data under different propagation conditions or the variance of distance estimates [4]. However, this method is highly dependent on specific environmental conditions; thus, its applicability is limited. The second type is based on geometric position estimation. This method determines the channel state by analyzing the historical location data of the mobile user or environmental data (such as geometric shapes and attenuation factors) [5]. This approach requires extensive computations and relies on rich prior information in order to accurately identify LOS and NLOS signals. The third type is based on channel statistics [6]. It mainly uses the statistical information of the received multipath components to identify LOS and NLOS, including parameters such as mean, standard deviation, root-mean-square delay spread (RMS-DS), skewness, kurtosis, etc. [7,8]. Reference [9] extracts features such as mean values, standard deviation, skewness, kurtosis, and Rician K-factor from the received signal strength of Wi-Fi signals as channel parameters for LOS and NLOS identification. Reference [10] analyzes channel impulse responses and extracts 18 features to identify NLOS conditions, but this method does not consider the correlations between feature parameters. Reference [11] combines the sum of peak time and rise time, along with the number of undetected peaks, as feature parameters for identification. However, if the peak time and rise time differ significantly from expectations or the threshold is chosen incorrectly, this can easily lead to identification errors.

In recent years, with the rapid development of deep neural networks, the multilayer neural networks utilized in deep learning have also been widely used to identify LOS and NLOS propagation conditions. These methods use CIR data as a system input and complete feature extraction through self-learning models. CIR represents the signal’s time-domain response after propagation through a channel, providing detailed information about the multipath propagation effects, which is crucial for distinguishing between LOS and NLOS conditions. Reference [12] proposes a method that uses reversible transformation for denoising and combines convolutional neural networks (CNNs) to identify NLOS signals, achieving a recognition accuracy of up to 81.68%. Reference [13] uses raw CIR as input, combining convolutional neural networks and long short-term memory (LSTM) models for training and testing, achieving a recognition accuracy of up to 82.14%. Reference [14] proposes a method that uses raw CIR and its Fourier transform’s real and imaginary parts as inputs, employing a three-channel convolutional neural network combined with a bidirectional long short-term memory (BiLSTM) network for identification. This method, validated with the public dataset from the Horizon 2020 project eWINE, outperforms methods using LSTM or CNN-LSTM alone. Reference [15] proposes a deep learning-based UWB NLOS/LOS classification algorithm called FCN–Attention. This algorithm uses a fully convolutional network (FCN) to enhance feature extraction capabilities and combines an attention mechanism to strengthen data feature description, improving classification accuracy.

In the existing research on NLOS identification, many scholars have focused on optimizing the structure of deep learning networks to improve the identification accuracy of NLOS and LOS signals. However, the direct impact of model parameters (such as learning rate, weight initialization, regularization parameters, and batch size) on the model training process and the final identification performance is often overlooked. Although some studies have made progress in the optimization algorithms of UWB indoor positioning system (such as the bidirectional ranging algorithm and C-Taylor hybrid weighting algorithm) and improved the positioning accuracy through the adaptive robust Kalman filter algorithm [16], the feasibility of using UWB positioning systems to track sows in a complex environment has also been verified [17]. At the same time, the improved deep residual network performs well in fault diagnosis under unbalanced data conditions [18]. However, in practical applications, the signal transmission under non-line-of-sight (NLOS) conditions is affected by the reflection and blocking of obstacles, resulting in signal path lengthening and distortion. This significantly affects the accuracy of UWB positioning. Achieving high-precision and efficient NLOS identification remains extremely challenging. Therefore, this paper selects the basic algorithm for neural network training—the backpropagation (BP) algorithm—as the classifier for NLOS identification and proposes an NLOS identification model based on an improved Snake Search Algorithm-optimized BP neural network. By integrating various improvement strategies, this paper combines Logistic and Tent chaotic mapping to initialize the population, introduces a subtractive optimizer algorithm to update the positions in the Snake exploration phase, and incorporates dynamic development probability to expand the search space, thereby improving the Snake Search Algorithm. The improved Snake Search Algorithm is used to optimize the weights and thresholds of the BP neural network, and the model is validated based on the collected NLOS classification dataset.

## 2. Preliminaries

### 2.1. BP Neural Network

**Figure 1:**
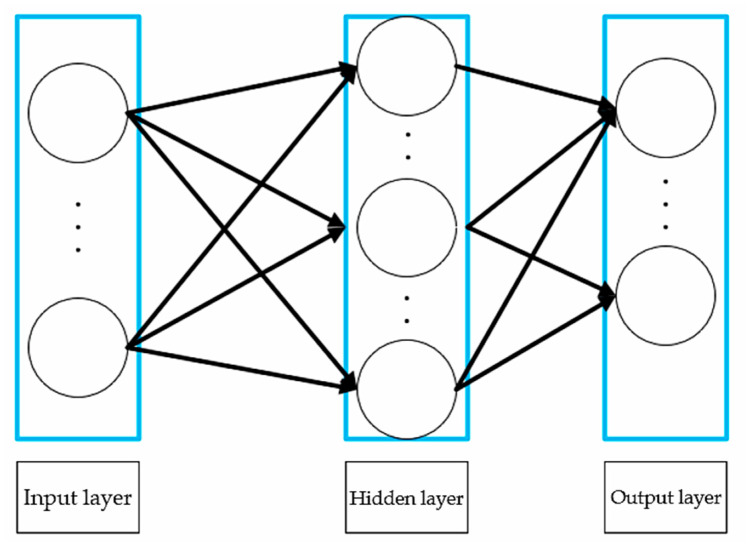
BP neural network model.

A backpropagation (BP) neural network is a type of artificial neural network widely used in supervised learning [19]. The BP neural network is composed of multiple layers of neurons, including an input layer, hidden layers, and an output layer, adopting a layered structure to facilitate the effective transmission of data between layers. Neurons in each layer are connected and weighted with specific weights to the neurons in the next layer. This structure enables efficient information transmission and transformation during data processing and feature extraction. Its structure is shown in Figure 1. The BP algorithm is widely applied due to its efficiency and practicality in solving nonlinear mapping problems between engineering inputs and outputs [20]. However, the performance of traditional BP neural networks heavily depends on the configuration of multiple hyperparameters, such as learning rate, regularization parameters, and weight initialization. Therefore, the introduction of intelligent optimization algorithms for global searches can determine the optimal initial weight and threshold configurations. This not only significantly enhances the network’s ability to solve complex problems, but also reduces its dependency on random initialization [21].

### 2.2. Snake Optimizer

The Snake Optimizer (SO), proposed by Fatma A. Hashim in 2022, is a novel intelligent optimization algorithm inspired by the unique mating behaviors of snakes [22]. It demonstrates superior global optimization capabilities compared to traditional algorithms, such as the Particle Swarm Optimizer (PSO) and Grey Wolf Optimizer (GWO). The design of the Snake Optimizer is deeply influenced by the mating behaviors of snakes, which are affected by factors like temperature and food availability. Specifically, in nature, snakes are inclined to engage in mating activities when the environmental temperature is suitable and food is abundant. Conversely, when conditions are unfavorable, snakes prioritize searching for food or utilize existing food resources. The detailed process of the algorithm is as follows.

1. Initialization and population partitioning

Similar to other metaheuristic algorithms, the SO algorithm sets the initial solutions through a random initialization process, as shown in Equation (1):

(1) $$X_{i}=X_{min}+rand\times \left(X_{max}-X_{min}\right),$$

where $X_{i}$ represents the position of the i-th solution, rand is a random number between 0 and 1, and $X_{max}$ and $X_{min}$ represent the upper and lower bounds of the problem search space, respectively. The initial population is roughly divided into male and female groups in a ratio of approximately 1:1. The search process of the SO algorithm is mainly influenced by temperature (Temp) and the quantity of food (Q), defined in Equation (2) as shown:

(2) $$\{\begin{array}{c} Temp=\exp\left(\frac{-L}{T}\right)\\ Q=c_{1}\times \exp\left(\frac{L-T}{T}\right) \end{array},$$

In Equation (2), *L* represents the current iteration number, *T* denotes the maximum number of iterations, and $c_{1}$ is a predefined constant. During each iteration of the algorithm, by evaluating the performance of each individual within the groups, the best-performing male and female individuals and their positions can be identified.

2. Exploration stage

When the quantity of food in the SO algorithm is below a specific threshold, *TH*_1_, the algorithm enters the exploration phase. In this phase, food resources in the environment are scarce, and so individuals randomly select the positions of other individuals for food searching, updating their positions and fitness as shown in Equation (3):

(3) $$\{\begin{array}{c} X_{i,m}\left(t+l\right)=X_{r}\left(t\right)\pm c_{2}\times A\times \left(\left(X_{max}-X_{min}\right)\times r+X_{min}\right)\\ A=\exp\left(\frac{-f_{r}}{f_{i,m}}\right) \end{array},$$

where $X_{r}$ is the position of a randomly selected individual snake, $c_{2}$ is a constant, A represents the food-finding ability of the snake, and $f_{r}$ and $f_{i,m}$ represent the fitness values of positions $X_{r}$ and $X_{i,m}$, respectively.

3. Development phase

When Q exceeds the threshold $TH_{1}$, the algorithm shifts to its exploitation phase. Additionally, if Temp is higher than another threshold, $TH_{2}$, this indicates that the environment is in a high-temperature state. Under such conditions, the snake population will primarily focus on finding and utilizing the currently discovered food resources, i.e., performing local search and optimization around the known optimal solution. In this high-temperature and environment with abundant food, the position update mechanism of the snake population is as shown in Equation (4):

(4) $$X_{i,j}\left(t+l\right)=X_{food}\pm c_{3}\times Temp\times r\times \left(X_{food}-X_{i,j}\left(t\right)\right),$$

where $X_{i,j}$ is the position of the snake individual (male or female), $X_{food}$ is the optimal position of the snake individual, r is a random number in the range [0, 1], and $c_{3}$ is a constant. When the temperature is less than or equal to the threshold $TH_{2}$, the SO algorithm introduces combat modes and mating modes to simulate the survival behavior of snakes in nature. The trigger for these two modes depends on the value of the random number: when the random number is less than 0.6, the snake individual adopts combat mode; otherwise, it enters mating mode. This design aims to increase the algorithm’s diversity and improve its optimization ability. In combat mode, snake individuals update their positions to enhance their ability to compete for resources. Both male and female snake individuals use the same position update and combat capability calculation, as shown in Equation (5):

(5) $$\{\begin{array}{c} X_{i,m}\left(t+l\right)=X_{i}\left(t\right)+c_{3}\times F\times r\times \left(Q\times X_{best,f}-X_{i,m}\left(t\right)\right)\\ F=\exp\left(\frac{-f_{best,m}}{f_{i}}\right) \end{array},$$

where $X_{best,f}\;$represents the best position in the female snake group, and $f_{best,m\;}\;$represents the fitness value of that position. $f_{i}\;$is the fitness value of the snake individual, and $c_{3}$ and r are control factors and a random number within the range [0, 1], respectively. Here, the combat capability F reflects the degree of advantage of individuals possess in resource competition, encouraging the algorithm to explore areas with higher fitness. In mating mode, both male and female snake individuals adopt the same position update to enhance mating opportunities, and the mechanism is shown in Equation (6):

(6) $$\{\begin{array}{c} X_{i,m}\left(t+l\right)=X_{i,m}\left(t\right)+c_{3}\times M\times r\times \left(Q\times X_{i,f}-X_{i,m}\left(t\right)\right)\\ M=\exp\left(\frac{-f_{i,f}}{f_{i,m}}\right) \end{array},$$

where M represents the mating capability of the individual, and $f_{i,m}$ and $f_{i,f}$ are the fitness values of the i-th male and female positions, respectively. When mating successfully results in hatched eggs, the algorithm adopts a natural selection strategy by selectively replacing the worst-performing male and female individuals to simulate the natural selection process. The replacement mechanism for both males and females is as shown in Equation (7):

(7) $$X_{worst,m}=X_{min}+rand\times \left(X_{max}-X_{min}\right).$$

## 3. Improved Snake Optimizer

### 3.1. Population Initialization

The distribution of the initial population plays a crucial role in intelligent optimization algorithms, directly affecting the algorithm’s ability to explore the solution space and efficiently find the optimal global solution. In the Snake Optimizer algorithm, although the initial population is generated randomly, this randomness does not always ensure population diversity. If the randomly generated initial individuals are too concentrated or unevenly distributed, the algorithm might miss key search areas at the initial stage, leading to reduced exploration efficiency and potentially affecting the quality of the final solution. Chaos theory provides a powerful tool for analyzing and predicting complex behaviors in dynamic systems. Chaotic mapping is widely applied in the population initialization of optimization algorithms due to its unique properties, which helps to enhance function optimization efficiency. Random number sequences generated by chaotic mapping achieve uniform distributions in the solution space, thus avoiding the omission of certain areas in the solution space and enhancing the algorithm’s global search capability [23].

Logistic mapping and Tent mapping are two widely used chaotic models [24]. These models are often used for population initialization to enhance algorithms’ performances. Logistic mapping is a nonlinear recursive mapping technique primarily used to simulate biological population growth. It exhibits rich dynamic behavior in its simple form, but its chaotic behavior is mainly concentrated within specific parameter ranges. The Logistic mapping function is shown in Equation (8):

(8) $$X_{k+1}=\theta X_{k}\left(l-X_{k}\right),$$

where $X_{k}$ usually takes values within [0, 1]. The parameter θ is a Logistic parameter, typically ranging from [0, 4]. The Tent chaos is a piecewise linear one-dimensional mapping known for its clear linear segments and rapid transition to a chaotic state. Tent mapping can easily reach a chaotic state across the entire parameter space, although its chaotic characteristics are relatively simple. The Tent chaotic function is shown in Equation (9), where *a*∈(0,1) is the chaotic parameter:

(9) $$x_{t+1}=\{\begin{array}{cc} \frac{x_{t}}{a} & 0\leq x_{t}\leq a\\ \frac{1-x_{t}}{1-a} & a<x_{t}\leq 1 \end{array}.$$

**Figure 2:**
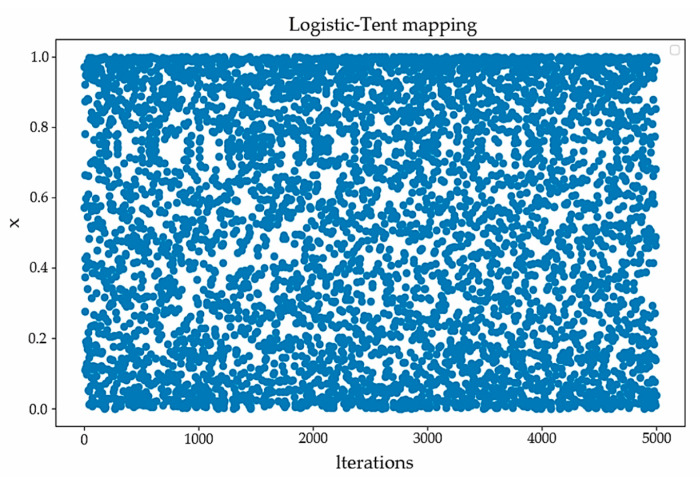
Logistic–Tent chaotic mapping.

The improved SO algorithm utilizes a combination of Logistic and Tent (LT) chaotic mappings for population initialization. This method integrates the complex nonlinear characteristics of the Logistic map with the strong chaotic properties of the Tent map, thereby enabling the system so that it exhibits chaotic behavior over a wider parameter range. The LT chaotic mapping function is shown in Equation (10):

(10) $$x_{t+1}=\{\begin{array}{c} \left(a\times x_{t}\times \left(1-x_{t}\right)+\left(4-a\right)\times \frac{x_{t}}{2}\right)\mathrm{mod}1\;\;\;\;x_{t}<0.5\\ \left(a\times x_{t}\times \left(1-x_{t}\right)+\left(4-a\right)\times \left(\frac{1-x_{t}}{2}\right)\right)\mathrm{mod}1\;\;\;\;x_{t}\geq 0.5 \end{array},$$

where a∈(0, 4) is a constant. Figure 2 illustrates the distribution of the Logistic–Tent mapping initialization after 5000 iterations.

### 3.2. Integration of SABO Update Strategy

To address the issue of a slow initial optimization speed in the snake optimization algorithm and enhance its global search capability and local search accuracy, this paper proposes an optimization approach by introducing the subtraction-average-based optimizer (SABO) update mechanism. SABO [25] is inspired by fundamental mathematical concepts, including averages, differences in search agent positions, and differences in the signs of objective function values. This method updates agent positions by describing the differences between search agents, as shown specifically in Equation (11):

(11) $$\bar{A}=\mathrm{sign}\left(F\left(A\right)-F\left(B\right)\right)\left(A-\vec{v}\times B\right),$$

where $\vec{v}$ is a vector of dimension *m*, with elements generated by random numbers within the interval [1, 2]. F(A) and *F*(*B*) represent the objective function values of search agents A and B, respectively, while the sign function is the sign function.

The SABO algorithm is renowned for its concise concept and rapid optimization capability, exhibiting an almost linear optimization curve throughout the optimization process. This indicates its ability to avoid significant deceleration or stagnation at various stages of the algorithm, effectively preventing it from falling into local optima. Based on this characteristic, this paper integrates the update strategy of the SABO algorithm into the SO algorithm, replacing the original Equation (3), as shown specifically in Equation (12):

(12) $$X_{i}^{new}=X_{i}+{\vec{r}}_{i}\times \frac{1}{N}\overset{N}{\underset{j=1}{\sum }}\left(X_{i}-vX_{j}\right),i=1,2,\ldots ,N.$$

This strategy is introduced with the aim of leveraging the efficient global search capability of SABO to enhance the speed and efficiency of the SO algorithm during the early stages of searching. Simultaneously, by finely adjusting the update mechanism of search agents, we improve the algorithm’s local search accuracy in the later iterations. This strategy addresses the issue of slow optimization speed in the early stages of the SO algorithm while maintaining the stability and continuity of the algorithm throughout the entire search process, further enhancing its performance in complex optimization problems.

### 3.3. Dynamic Development Factor Based Update

The fixed development probability during the developmental phase of the Snake Algorithm cannot flexibly adjust the balance between exploration and exploitation at different stages, which may lead to excessive focus on local regions in the early stages of the search or ineffective convergence to the global optimum in the later stages. Therefore, unlike in the above development phase, this paper proposes the concept of a dynamic development probability $TH_{2}$ to replace the traditional fixed development probability. The core idea of this dynamic development probability is to adaptively adjust the development probability based on the current state of the algorithm and search history in order to better balance the relationship between exploration and exploitation, as shown in Equation (13).

(13) $$TH_{2}=0.3-c\left(\frac{l}{T}-1\right),$$

**Figure 3:**
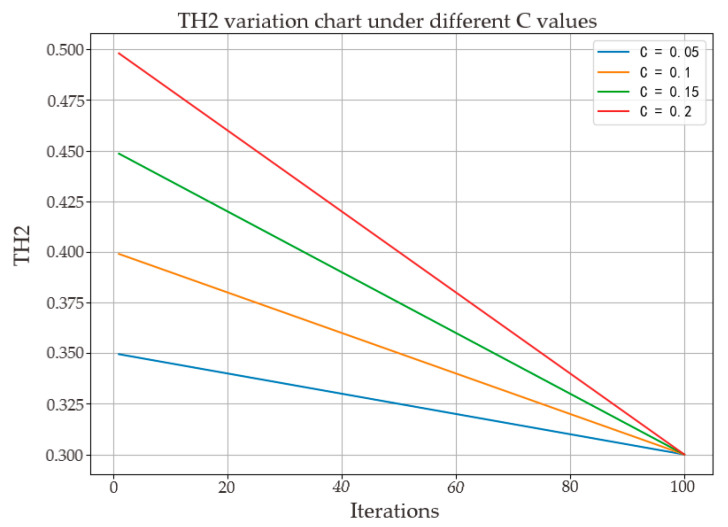
The variation law of *TH*_2_ with dynamic development factors.

In the equation, l represents the current iteration count, T is the maximum iteration count, and c is a constant dynamic development factor. $TH_{2}$ varies according to the dynamic development factor, as illustrated in Figure 3, where T is set to 100.

From the graph, it can be observed that as the value of c increases, the rate of decrease in $TH_{2}$ also increases. This phenomenon reveals a potential strategy: by dynamically adjusting the development probability $TH_{2}$, the snake optimization algorithm can effectively adopt different search strategies at different stages of the algorithm, thereby improving its search efficiency and solution accuracy. This adaptive adjustment mechanism ensures that the algorithm can conduct extensive global searches in the early iterations and focus on fine local searches around the global optimum in the later iterations.

### 3.4. LTSSO-BP Neural Network NLOS Training Model

**Figure 4:**
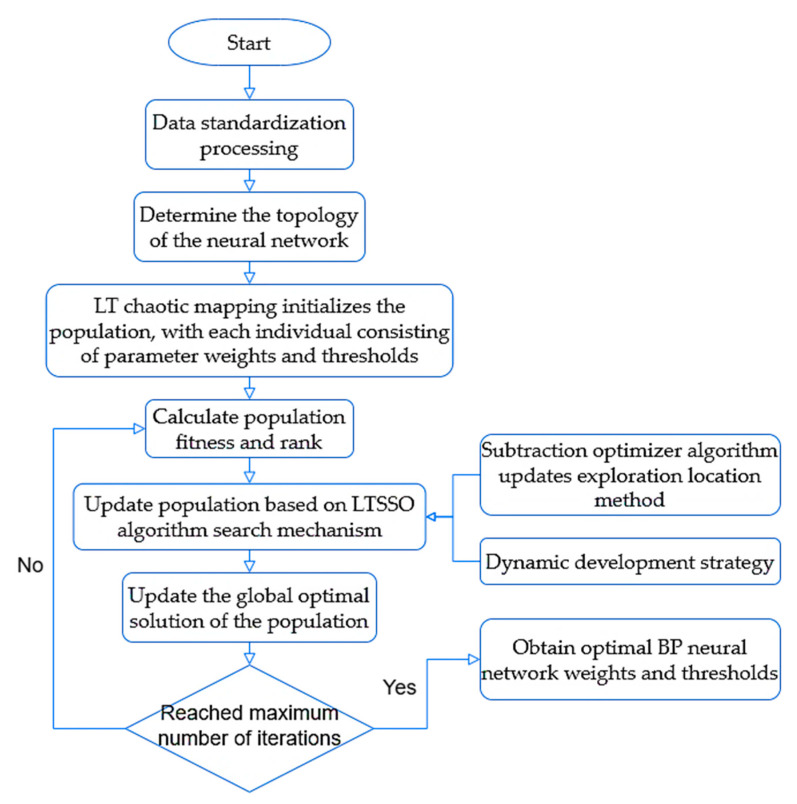
LTSSO-BP network flowchart.

Due to the inherent randomness in the initial weights and thresholds of the BP neural network, achieving global optimization becomes challenging, leading to significant fluctuations in the accuracy of prediction results [26]. Therefore, in this study, we employ the Snake Search Algorithm to optimize the weights and thresholds of the BP neural network. Additionally, considering the tendency of the Snake Search Algorithm to converge to local optima, we utilize an improved Snake Search Algorithm to optimize the weights and thresholds of the BP neural network, thus constructing a Logistic–Tent SABO Snake Optimizer-BP (LTSSO-BP) neural network prediction model, as illustrated in Figure 4. 

The specific steps include the following:

Step 1: Set up the relevant parameters of the LTSSO algorithm, including the population size, adjustment factor, and maximum number of iterations, and initialize the population using the LT chaotic mapping distribution.

Step 2: Determine the range of weights and thresholds, train the BP neural network based on the training set, and use the accuracy of the BP classifier as the fitness function to calculate the fitness of all individuals in the population.

Step 3: Determine whether the snake individuals enter the exploration phase or the exploitation phase based on the amount of food (T) and the iteration temperature (Q). In the exploration phase, update individual positions using the subtraction optimization algorithm formula; in the exploitation phase, determine whether snake individuals are in battle mode or mating mode based on dynamic development probability and update the corresponding formula to update individual positions.

Step 4: Check if the maximum number of iterations has been reached. If so, output the optimal parameter combination; if not, return to step 3 to continue iterating.

Step 5: After obtaining the optimal weight and threshold combination, retrain the BP model based on the training set.

Step 6: Test the trained model on the test set and evaluate the model’s performance.

## 4. Experiment and Result Analysis

### 4.1. Experimental Dataset

#### 4.1.1. Data Collection Equipment

**Figure 5:**
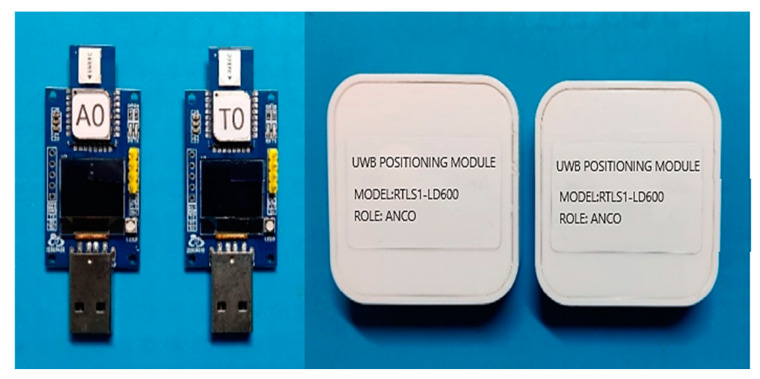
UWB positioning module for positioning base stations and tag devices.

In this study, the HR-RTLS1 series ULM1 devices were used for measurements. The setup included base stations labeled with the prefix ‘A’ and tags labeled with the prefix ‘T’, as shown in Figure 5. ULM1 devices are low-cost hardware that integrate UWB transceivers and additional inertial sensors. These devices are popular in indoor positioning applications and can easily be interfaced with Arduino platforms or standalone computers for conducting wireless channel measurements.

The ULM1 devices utilize the DW1000 wireless RF chip from Decawave. This chip adheres to the IEEE 802.15.4-2011 communication standard [27] and employs a two-way ranging (TOF/DS-TWR) algorithm to estimate the distance between the base stations and tags. This enables the system to achieve high-precision positioning of target objects, with an accuracy within ±10 cm.

#### 4.1.2. Data Collection Design

**Figure 6:**
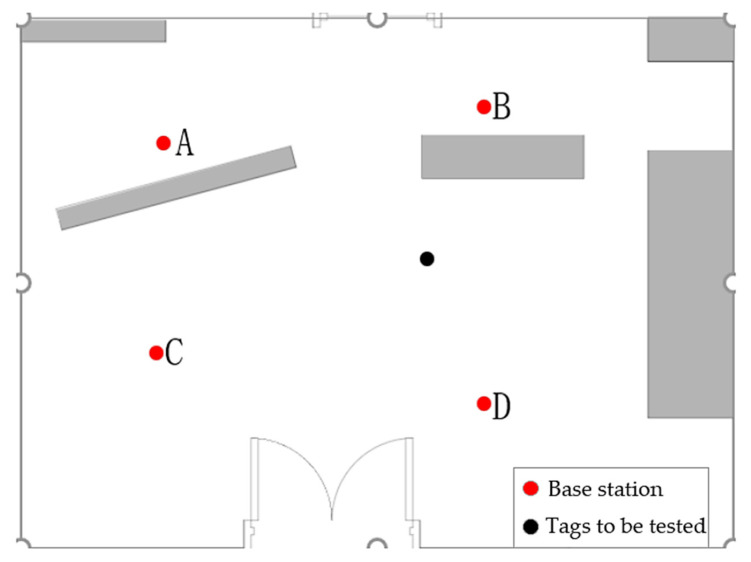
Schematic diagram of indoor data collection layout.

The dataset used in this study was collected in indoor environments using The DW1000 wireless radio frequency chip is manufactured by Decawave, a company based in Dublin, Ireland, covering channel impulse response (CIR) information under both non-line-of-sight (NLOS) and line-of-sight (LOS) conditions. Figure 6 illustrates the layout of the indoor data collection site, where the red dots represent the preset positions of the localization base stations, and the black dots represent the fixed positions of the tags designated for testing. To record data under different line-of-sight conditions, the base station devices were placed at locations A, B, C, and D to capture measurement results under NLOS and LOS conditions. Under these two propagation conditions, 2000 measurements were performed in each environment. 

#### 4.1.3. Data Collection

The DW1000 RF chip outputs multiple types of information during positioning. These include signal parameters such as the received Pulse Repetition Frequency (PRF), detection thresholds, and possibly a set of channel impulse response (CIR) samples. To distinguish between LOS and NLOS conditions, the collected data were labeled accordingly: LOS propagation is labeled as 0, while NLOS propagation is labeled as 1. The data items of the NLOS classification dataset are shown in Table 1.

### 4.2. Measurement Data Analysis

#### 4.2.1. Noise-Related Parameter Analysis

**Figure 7:**
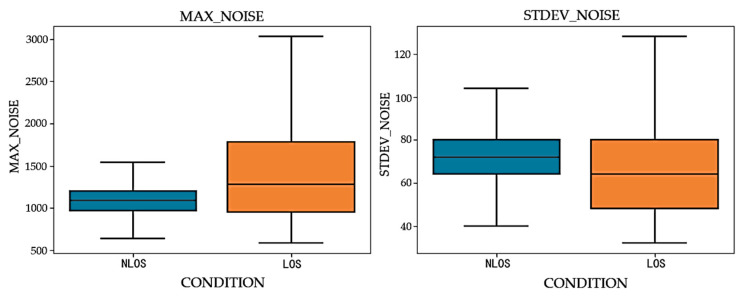
Performance of noise parameters in LOS and NLOS environments.

Based on the data collected under different visibility conditions, the distribution of maximum noise and noise standard deviation is plotted, as shown in Figure 7. From the box plot, it can be observed that in both non-line-of-sight (NLOS) and line-of-sight (LOS) environments, the parameters of maximum noise and noise standard deviation exhibit a high degree of similarity.

#### 4.2.2. Signal Strength Parameter Analysis

Signal strength parameters have a decisive impact on the performance evaluation of wireless communication systems. During this data collection process, four core signal strength parameters were analyzed: fpAmp1, fpAmp2, fpAmp3, and CIR_PWR.

- fpAmp1 represents the amplitude of the first path channel impulse response sampling point, directly reflecting the strength of the signal at the initial reception point and providing an intuitive measure for assessing the initial reception quality of the signal;
- fpAmp2 and fpAmp3, as the amplitudes of the subsequent two CIR sampling points following fpAmp1, demonstrate the immediate changes in the signal after the first path detection, offering insights into the signal’s propagation and subsequent multipath effects;
- CIR_PWR measures the maximum variation of signal strength within a specific time window, revealing the dynamic range of the signal and its stability in complex environments.

**Figure 8:**
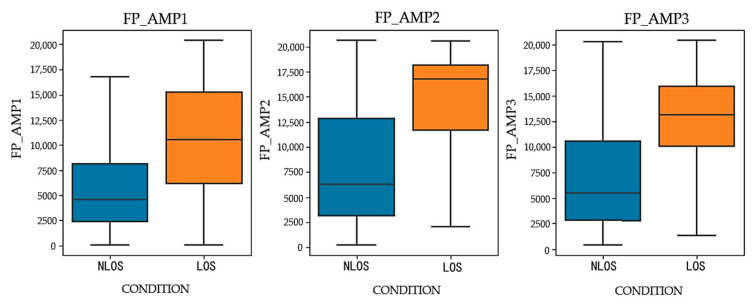
Path amplitude in LOS and NLOS environments.

**Figure 9:**
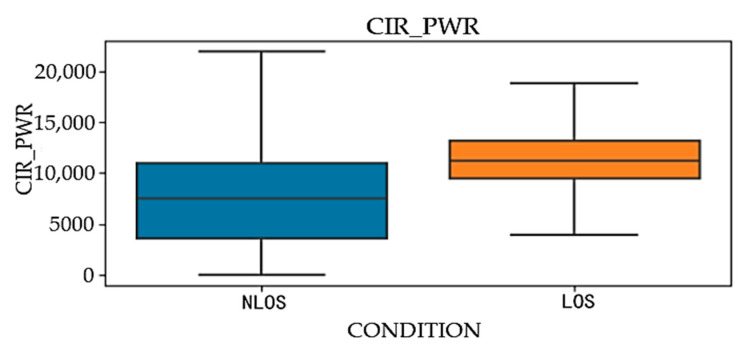
CIR_PWR in LOS and NLOS environments.

The distribution of these four parameters under different propagation environments is shown in Figure 8 and Figure 9. The analysis of these figures leads to the conclusion that in line-of-sight (LOS) environments, the median values and interquartile ranges of these parameters are significantly higher than those in non-line-of-sight (NLOS) environments.

#### 4.2.3. Channel Impulse Response (CIR) Data Analysis

**Figure 10:**
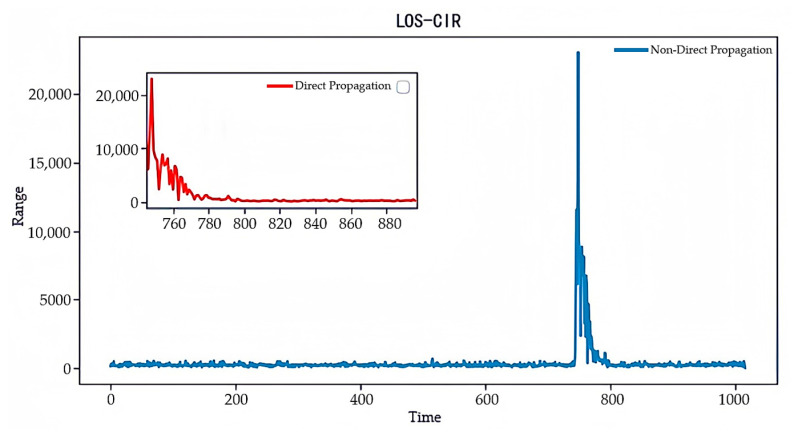
CIR waveform amplitude under LOS conditions.

**Figure 11:**
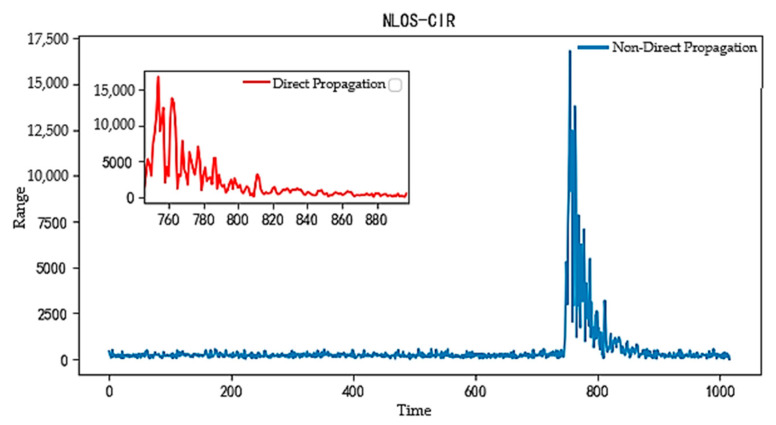
CIR waveform amplitude under NLOS conditions.

Channel impulse response (CIR) data [28] are among the key parameters used to describe channel characteristics in wireless communication systems. These data record the time-domain response of signals during propagation, reflecting information such as delay, amplitude, and phase as the signal arrives at the receiver through different paths. Figure 10 and Figure 11, respectively, show the CIR plots under LOS and NLOS conditions. By comparing these two figures, we can clearly observe that the UWB signal’s channel impulse response exhibits significant differences between direct and non-direct propagation conditions. Under LOS conditions, the signal is transmitted directly to the receiver, resulting in slower attenuation and higher peak values in the CIR waveform. This makes the direct path signal more frequent and easier to identify. Conversely, under NLOS conditions, signal attenuation increases due to obstacles, resulting in lower peak values in the waveform. The collected CIR data typically contain 1016 sampling points, but often include unnecessary, redundant sampling points. These redundant sampling points not only do not contribute to network performance but may also introduce noise, negatively affecting system performance. According to the literature [29], about 152 CIR sampling points are sufficient to capture the majority of the environmental propagation characteristics.

### 4.3. Model Related Parameters

The collected dataset was divided into two parts: 80% for model training and 20% for model testing. Then, the performance of BP, PSO-BP, SO-BP, and LTSSO-BP neural network models in NLOS classification prediction problems was compared. The input data for prediction were 152-dimensional, and the output data were 1-dimensional. Each neural network model was iterated 1000 times with a learning rate of 0.1. Both tanh(x) and sig(x) were used as activation functions for the hidden and output layers, respectively.

Determining the number of nodes in the hidden layer of the BP neural network is a crucial step in network design. The choice of hidden layer nodes directly affects the network’s learning ability, generalization ability, and computational complexity. When determining the number of hidden layer nodes, an empirical formula can be used to obtain an approximate range, and then the number of hidden layer nodes can be selected within this range to minimize the root-mean-square error through trial and error. The empirical formula is as follows:

(14) $$m=\sqrt{n+l}+z,$$

where *m* is the number of neurons in the hidden layer; *n* is the number of neurons in the input layer; *l* is the number of neurons in the output layer; and *z* is a fixed constant, z∈ (0,10).

Through trial and error, the final number of hidden layer nodes is determined to be 13.

### 4.4. Performance Evaluation Indicators

The experimental platform uses a 64-bit Windows 10 operating system, Matlab R2023b, equipped with a 2.4 GHz CPU and 8 GB of memory. This study evaluates the classification performance of the classifier for NLOS classification experiments using three metrics: accuracy, recall, and F1 score. To assess the classification method of the LTSSO-BP classifier, the detection results can be categorized into four types [30]:

1. True positives (TP): cases where the classifier correctly identifies NLOS.
2. True negatives (TN): cases where the classifier correctly identifies LOS.
3. False positives (FP): cases where the classifier incorrectly identifies LOS as NLOS.
4. False negatives (FN): cases where the classifier incorrectly identifies NLOS as LOS.

### 4.5. Result Analysis

**Figure 12:**
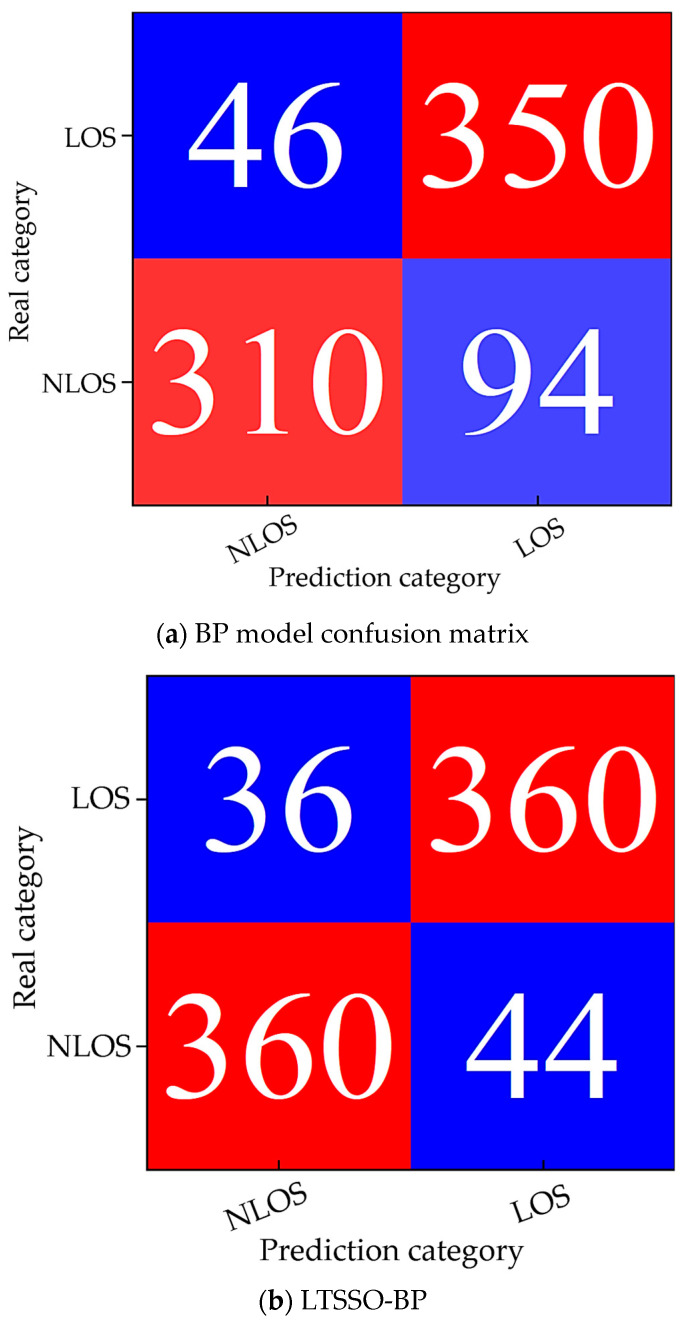
(**a**) BP model confusion matrix; (**b**) LTSSO-BP.

The confusion matrix plots for the BP model and LTSSO-BP model, respectively, are shown in Figure 12. It can be observed that the LTSSO-BP model exhibits a significant improvement in the classification accuracy of signal propagation states compared to the original BP model, which generally meets the basic requirements for accurate classification.

The classification accuracy, recall rate, and F1 score for the four models are summarized in Table 1. The results indicate that the optimized LTSSO-BP model achieves the best classification performance. From Table 2, it can be seen that when, only using the BP neural network for classification, the classification accuracy is relatively low. The inclusion of optimization algorithms in the PSO-BP and SO-BP models leads to a noticeable improvement in classification accuracy. In the LTSSO-BP model, further optimization of the Snake Search Algorithm results in final classification accuracy, recall rate, and F1 score values of 90%, 91.41%, and 90.25%, respectively, indicating that this model achieves the highest classification accuracy. 

### 4.6. Advantages and Disadvantages

This paper is devoted to researching NLOS identification and error suppression technology in wireless positioning. Although some progress has been made in NLOS identification accuracy and NLOS ranging error prediction, there are still some shortcomings in this paper, and further research and discussion are still needed.

- The constructed data sets for NLOS classification and error correction are built from data sets collected in a single indoor room. In order to improve the effectiveness and applicability of NLOS classification and error suppression algorithms, we can consider adding data sets from different indoor scenes in the future.
- This paper optimizes the parameters of the BP model by improving the snake optimization algorithm, effectively improving the classification ability of the model. However, this algorithm increases the computing resource consumption and training time. To this end, subsequent research will focus on designing more targeted algorithms to solve these problems. On the one hand, more efficient search strategies and dynamic tuning mechanisms will be explored to reduce the computational resource requirements of the algorithm while maintaining good performance. In addition, advanced parallel computing techniques, such as distributed computing, are introduced to accelerate the training process of the algorithm.

## 5. Future Direction

Future research will explore the integration of additional chaotic mapping strategies and optimization algorithms to further refine the model. Expanding the dataset to include diverse indoor environments and real-time testing will be crucial for validating the model’s robustness and applicability. Additionally, exploring hybrid models that combine deep learning techniques with the improved SSA may offer further improvements in signal classification accuracy and positioning reliability. We plan to validate the algorithm’s performance under dynamic label positions and varying NLOS/LOS conditions by simulating moving tags and unstable anchor conditions. Further experiments will include testing the effect of different obstacle materials on signal propagation to evaluate the algorithm’s performance under various environmental conditions.

In addition, this study plans to introduce a 10-fold cross-validation method in future experiments to comprehensively verify the performance of the algorithm and report the standard deviation values of the results. This method is widely used in the field of machine learning and pattern recognition, and is suitable for evaluating the performance of algorithms under different data partitions. Due to the current limitations of computing resources and time, we cannot immediately conduct these additional experiments, but in future research, we will give priority to solving these problems and ensuring the integrity of the experiment and the credibility of the data.

## 6. Conclusions

This study presents an LTSSO-BP model for classifying UWB signal propagation states. By enhancing the Snake Search Algorithm with Logistic–Tent chaotic mapping for population initialization, subtraction optimization for exploration, and dynamic probability adjustment, we improve the model’s classification performance. Our LTSSO-BP model outperforms BP, PSO-BP, and SO-BP models in classification accuracy and generalization, achieving the highest accuracy, recall, and F1 score among the evaluated models. These results highlight the effectiveness of our approach in enhancing UWB indoor positioning accuracy by effectively identifying NLOS and LOS signals.

## Figures and Tables

**Table 1:** NLOS classification dataset.

Data Item	Meaning
NLOS	1 represents NLOS, 0 represents LOS
FP_IDX	Index of the first path of the received signal
FP_AMP	Path amplitude
STDEV_NOISE	Noise standard deviation
CIR_PWR	Total power of the channel impulse response
MAX_NOISE	Maximum noise value
RXPACC	Received preamble symbols
CH	Channel number
FRAME_LEN	Data frame length
PREAM_LEN	Preamble length
BITRATE	Bit rate
CIR	Absolute value of the channel impulse response, length of 1016 ns

**Table 2:** Comparison of performance of different algorithms.

Algorithm	Accuracy	Recall	F1
BP	82.5%	87.08%	81.58%
PSO-BP	85.5%	88.71%	85.05%
SO-BP	87.25%	89.53%	87.02%
LTSSO-BP	90%	91.41%	90.25%

## Data Availability

The data used to support the findings of this study are available from the corresponding author upon request.
